# Cryopreservation in rhinoceros—Setting a new benchmark for sperm cryosurvival

**DOI:** 10.1371/journal.pone.0200154

**Published:** 2018-07-11

**Authors:** Robert Hermes, Thomas Bernd Hildebrandt, Frank Göritz

**Affiliations:** Leibniz Institute for Zoo and Wildlife Research, Berlin, Germany; Justus Liebig Universitat Giessen, GERMANY

## Abstract

At times when rhinoceros are fiercely poached, when some rhinoceros species are closer than ever to extinction, and when the scientific community is in debate over the use of advanced cell technologies as a remaining resort it is time to simplify and improve existing assisted reproduction techniques to enhance breeding and genetic diversity in the living populations under our care. Semen cryopreservation has been performed in all captive rhinoceros species with limited degree of success. Here we tested three freezing extenders, containing different cryoprotectants and various freezing rates for the cryopreservation of rhinoceros sperm from 14 bulls. In experiment I, semen from 9 bulls was used to determine the most suitable diluent, cryoprotectant and freezing rate for the successful cryopreservation of rhinoceros sperm. In experiment II, semen from 5 bulls was used to assess whether the removal of seminal plasma could further improve post thaw sperm quality following cryopreservation with conditions identified in Experiment I. Semen was diluted with Berliner Cryomedia, ButoCrio® or INRA Freeze®, packaged in 0.5 mL straws and frozen 3, 4, and 5 cm over liquid nitrogen (LN) vapour or directly in a dryshipper. It was found that semen extended with ButoCrio® (containing glycerol and methylformamide) and frozen 3cm over LN vapour provided the best protection to rhinoceros spermatozoa during cryopreservation. When pooled over treatments, total and progressive post thaw motility was 75.3 ± 4.2% and 68.5 ± 5.7%, respectively marking a new benchmark for the cryopreservation of rhinoceros sperm. Post thaw total and progressive motility, viability and acrosome integrity of semen diluted in ButoCrio® was significantly higher than semen extended in Berliner Cryomedia or INRA Freeze®. The removal of seminal plasma did not improve post thaw sperm survival (p > 0.05). In conclusion, the cryosurvival of rhinoceros spermatozoa was significantly improved when using a mixture of glycerol and methylformamide in combination with a fast freezing rate at 3 cm. These results describe a new protocol for the improved cryosurvival of rhinoceros spermatozoa and will enable a more successful preservation of genetic diversity between males, especially in donors whose spermatozoa may already be compromised prior to or during collection. The successful reduction of glycerol concentration in favour of methylformamide as a cryoprotectant could be a novel suggestion for the improvement of cryopreservation techniques in other wildlife species.

## Introduction

Polge and Rowson’s publication on the use of glycerol as a cryoprotectant for spermatozoa during the freezing process in 1952 marked a milestone in mammalian sperm cryopreservation [1]. As a proof of principle, semen frozen during these early days and thawed over 50 years later, still fertilized and produced live offspring in the early 2000’s [Polge, personal communication]. Decades on, freezing protocols have been further refined and as such, the use of frozen sperm is now preferred over fresh sperm for use in many domestic artificial insemination (AI) breeding programs, in some cases, achieving better results than natural breeding programs [2,3,4]. The ease of long term storage and distribution for AI, the preservation of valuable genetic lines and breeds and the ability to screen donors for infectious diseases prior insemination marks the success of sperm cryopreservation in domestic animal breeding programs. Forty years after the discovery of sperm cryopreservation over 95% of all bull semen is processed as frozen sperm [5] with single donors providing an estimate of 200.000 frozen AI doses per year [3] and only few countries still allowing the use of fresh semen for AI [6].

However, the transfer of this extensive body of knowledge on sperm cryopreservation to endangered wildlife species still encounters many challenges and difficulties [7]. In general, regular access to semen donors is one of the greatest challenges in wildlife species as it requires anaesthesia. Furthermore, the circumstances under which the semen sample is harvested, e.g. post mortem from the epididymis, post coital, by artificial vagina or by electroejaculation has a large influence on the presence, volume and composition of seminal plasma which has been shown to influence chilling, freezing and fertilizing properties of spermatozoa in a range of species [7,8,9]. As such, the development and refinement of high quality sperm cryopreservation techniques in wildlife species has been limited compared to other domestic species.

In rhinoceros, the first cryopreservation of semen was reported in a black rhinoceros by Platz et al. in 1979 [10]. Since then cryopreservation of rhinoceros’ sperm has been reported in white, black, Sumatran and Indian rhinoceros. Initial reports documented a post thaw motility of less than 30% in a few individuals of different species [11,12,13]. Since the discovery of the use of high-quality, sperm-rich fractions [14,15,16,17] and the use of standard equine or custom-made semen extenders containing up to 20% egg yolk, mean post thaw motility has increased to less than or equal 55%. Further studies in Indian and Sumatran rhinoceros, found no significant difference between glycerol and dimethyl sulfoxide as cryoprotectants [12,17] and a comparative study in white rhinoceros, suggested that large (8 mL / straw) volume freezing resulted in higher post thaw results compared to small (0.5 mL / straw) volume freezing [15]. Despite post thaw results being acceptable across rhinoceros species, cryopreserved sperm has been shown in principle to be fertile when used in white and Indian rhinoceros AI programs [14,18]. Low pregnancy rates after AI in Indian rhinoceros are hypothesised to be a consequence of general anaesthesia, insemination technique and AI timing rather than as the result of low post thaw semen quality [18].

Assisted reproduction technologies (ART) still seem greatly underutilised in rhinoceros’ reproduction management. At the same time, the Sumatran, Javan and northern white rhinoceros are highly endangered species for which ART may be the last resort to save them from extinction. The most drastic example in this regard is the northern white rhinoceros (*ceratotherium simum cottoni*). The three last individuals left are all infertile and can therefore be considered as living extinct [19]. Faced with this probable disaster, the scientific community has discussed whether cellular and advanced assisted reproductive technologies could save highly endangered species such as the northern white rhinoceros from extinction despite the very limited resources from the few individuals available for this undertaking [19]. Gametes and tissue cells preserved from two living and few deceased northern white rhinoceros drastically demonstrate the need for a long term, high quality gamete preservation strategy in endangered species. To date DMSO and glycerol are used for the cryopreservation of stallion spermatozoa, the domestic species closest related to the rhinoceros. Yet new cryoprotectants, including methylformamide or dimethylformamide, have been shown to yield superior post thaw results [20,21]. For further improvement of sperm cryopreservation in rhinoceros here, we aim to compare three different freezing extenders containing glycerol, DMSO or a combination of glycerol and methylformamide. Additionally, we will test whether seminal plasma, with its multitude of functions as an energy source, transport vehicle and regulator of capacitation or sperm storage may truly have a negative influence on the cryosurvial of rhinoceros spermatozoa similar of the boar, stallion or elephant [22].

## Methods and materials

### Ethics statement

This study was conducted on captive white, black and greater one-horned rhinoceros, species listed by the IUCN as vulnerable, critically endangered and near threatened, respectively. The study was approved by IACUC animal ethics committee of the Leibniz Institute for Zoo and Wildlife Research (permit number: 2014-02-02) and carried out in accordance with the German National Protection of Animals Act (last revision 15^th^ July, 2009).

### Animals and semen collection

Semen was collected by electroejaculation (Seager model 14, Dalzell USA Medical Systems, The Plains, VA, USA) from 14 adult, captive, mostly non-proven (10 / 14) rhinoceros bulls (n = 10 white, *ceratotherium simum*; n = 1 greater one-horned, *rhinoceros unicornis*; n = 3 black rhinoceros, *rhinoceros bicornis*) at 17 ± 3 years with superior native semen quality (Table 1) [23,24]. For semen collection by electrostimulation general anaesthesia in lateral recumbency was required. For this, 25 mg detomidine hydrochloride (Domidine^®^ 10 mg/mL, Eurovet Animal Health B.V., Bladel, The Netherlands) and 25 mg butorphanol (Torbugesic^®^ Vet 10 mg / mL, Zoetis B.V., Capelle a/d IJssel, The Netherlands) were injected intra muscular as a premedication. A combination of 150 mg ketamine hydrochloride (Ketamin 10% WDT, Henry Schein VET GmbH, Hamburg, Germany) and 1.8–2.7 mg ethorphine (Captivon, Wildlife Pharmaceuticals South Africa, Karino, South Africa) was injected intravenous into the ear vein after 20 minutes. Anaesthesia was antagonized by administration of 250 mg naltrexone hydrochloride (Trexonil^TM^, Wildlife Pharmaceuticals (PTY) Ltd., White River, South Africa) and 40 mg atipamezole hydrochloride (Atipam 5 mg / mL, Eurovet Animal Health B.V.). Half the reversal was given i.m. and the other half was given i.v. Animals were normal and alert two to three minutes after the antagonist was given.

**Table 1:** Volume and sperm parameters from 14 electro-ejaculated rhinoceros bulls.

Volume (ml)	Concentration (sperm x 10^6^/ml)	Total motility (%)	Progressive motility (%)	Live sperm (%)	Intact acrosomes (%)	Normal morphology (%)
6.0 ± 1.0	620.7 ± 102.1	90.3 ± 1.2	90.3 ± 1.2	92.4 ± 1.8	85.1 ± 6.4	84.0 ± 3.0

For electroejaculation, a custom-made electric probe, specifically designed for rhinoceros, was used for stimulation [25]. The stimulation probe expanded the lumen of the rectum providing maximum electric coupling of electrodes. Multiple sets of 2–3 electrical stimuli were applied with increasing voltage and amperage (5–15 V / 200–800 mA). Each set of stimulations was followed by manual massage of the pelvic and penile aspects of the urethra [13,16,17]. After each set of stimulations, semen was collected into foam-insulated 50 mL isotherm collection tubes and kept at body temperature until analysed. For analysis and cryopreservation, only the first 1–2 sperm-rich fractions were used. Subsequent fractions were discarded (Table 1).

### Experiment I: Testing freezing extenders and freezing rates

In experiment I, semen from 9 rhinoceros’ bulls (white: n = 7, black: n = 1, greater one-horned n = 1) was used. Concentration of spermatozoa in sperm-rich fractions was estimated immediately after collection using an improved Neubauer haemocytometer. The semen was then diluted to a concentration of ~100 x 10^6^ spermatozoa / mL using three different, pre-warmed (36.5° C) semen extenders: 1) Berliner Cryomedia (BC) [13,22,23,26], 2) BotuCrio®, (Nidacon, 431 37 Mölndal, Sweden) 3) INRA Freeze® (IMV Technologies, 61300 L'Aigle, France) which contained 6% DMSO, 1% glycerol + 4% methylformamide or 2.5% glycerol as cryoprotectants, respectively. Total (percentage of progressive + stationary motile sperm) and progressive (the percentage of sperms which cross at least two thirds of a field of view in a virtually progressive manner at a 200fold magnification) sperm motility were assessed in all three extender variants after initial dilution and after thawing. For this 10 μL aliquots were put on a pre-warmed slide and evaluated on a warm stage equipped stereomicroscope (Olympus C41, Olympus, Germany). Frozen-thawed spermatozoa were temperature challenged in a 36.5°C water bath for 3 h and assessed at 0 h, 1 h and 3 h post thaw. From the fresh diluted and the frozen thawed semen samples, 10 μL aliquots were fixed in 40 μL Hancock’s fixative for the assessment of acrosome integrity and sperm morphology [22]. Acrosomes were classified as intact versus modified or reacted (including completely detached acrosomes). Sperm morphology included a search for a wide range of abnormalities. In addition, 10 μL aliquots were stained for 30s in 40 μL of a one‐step eosin‐nigrosin stain (Sperm Vitalstain, Nidacon, 431 37 Mölndal, Sweden Schweden) for assessment of sperm vitality [27]. For the vitality assessment the percentage of live (unstained) and dead (stained) sperm cells was evaluated. For acrosome integrity, morphology and vitality a total of 100 spermatozoa were evaluated per slide. Throughout the study all samples were evaluated by the same experienced spermatologist.

Diluted semen was equilibrated for 15 minutes at room temperature before packaged into 0.5 mL straws and chilled for 45 minutes to 4°C (0.5°C / min). Straws of each extender treatment were frozen for 10 minutes at 3 cm, 4 cm, and 5 cm above liquid nitrogen (LN) vapour before being plunged into the liquid phase. Freezing rates for the respective distances over the liquid nitrogen vapour and the dryshipper were determined in advance using a temperature logger (PCE-T390, PCE Deutschland GmbH, 59872 Meschede, Germany) placed into a loaded 0.5 mL straw and placed onto a floating rack in the liquid nitrogen bath (Mintube, 84184Tiefenbach, Germany). Freezing rates from +4°C to -15°C at 3, 4 and 5 cm above LN surface were -11°C / min, -10°C / min and -8°C / min, respectively. Freezing rates from -15°C to– 100°C at 3, 4 and 5 cm above LN were -29°C / min, -19°C / min and -16°C / min, respectively. Manual seeding was performed 60s after freezing had started. To mimic uncontrolled, remote field conditions one straw of each variant was frozen in the bottom of a freshly charged dryshipper. Freezing rates in the dry shipper were -29°C / min from +4°C to -15°C and -67°C / min from -15°C to -100°C. All samples were thawed for 60s in 36.5°C water bath and kept at 36.5°C for 3 h post thawing.

### Experiment II: Removal of seminal plasma

In experiment II, the ejaculates from 5 bulls (white: n = 3, black: n = 2) were split in half. One half was diluted directly with ButoCrio containing 1% glycerol + 4% methylformamide to a concentration of ~100 x 10^6^ sperm cells / mL as described in experiment I. The other half of the ejaculate was centrifuged for removal of seminal plasma. For this, the ejaculate was placed in 15 ml conical tubes underlain with 1 mL of a high density gradient (Optiprep) to cushion the sperm cells and prevent mechanical damage from centrifugation [28]. The sample was centrifuged for 20 minutes at 1000g. After centrifugation the supernatant was discarded, the sperm rich-layer above the density gradient aspirated and re-suspended in ButoCrio containing 1% glycerol + 4% methylformamide to a final concentration of ~100 x 10^6^ sperm cells / mL. Both variants, with and without seminal plasma, were equilibrated for 15 minutes at room temperature before being packaged into 0.5 mL straws and chilled for 45 minutes to 4°C (0.5°C / min). Semen was frozen 3 cm above LN vapour, the freezing rate which provided best post thaw results in experiment I, before being plunged into LN. Semen parameters of the two variants were assessed as described for experiment one.

### Statistical analysis

Values for motility, vitality, acrosome integrity, and normal sperm morphology are reported as mean ± SEM. Repeated measures ANOVA was performed on paired values of different extenders frozen at the same freezing rate or of the same extender frozen at different freezing rates after all values had passed the Kolmogorov and Smirnov assumption test. Tukey-Kramer multiple comparisons test was performed as post-test. P values < 0.05 were considered significant.

## Results

### Experiment I

#### Comparison of post thaw sperm quality between freezing extenders

**Fig 1:**
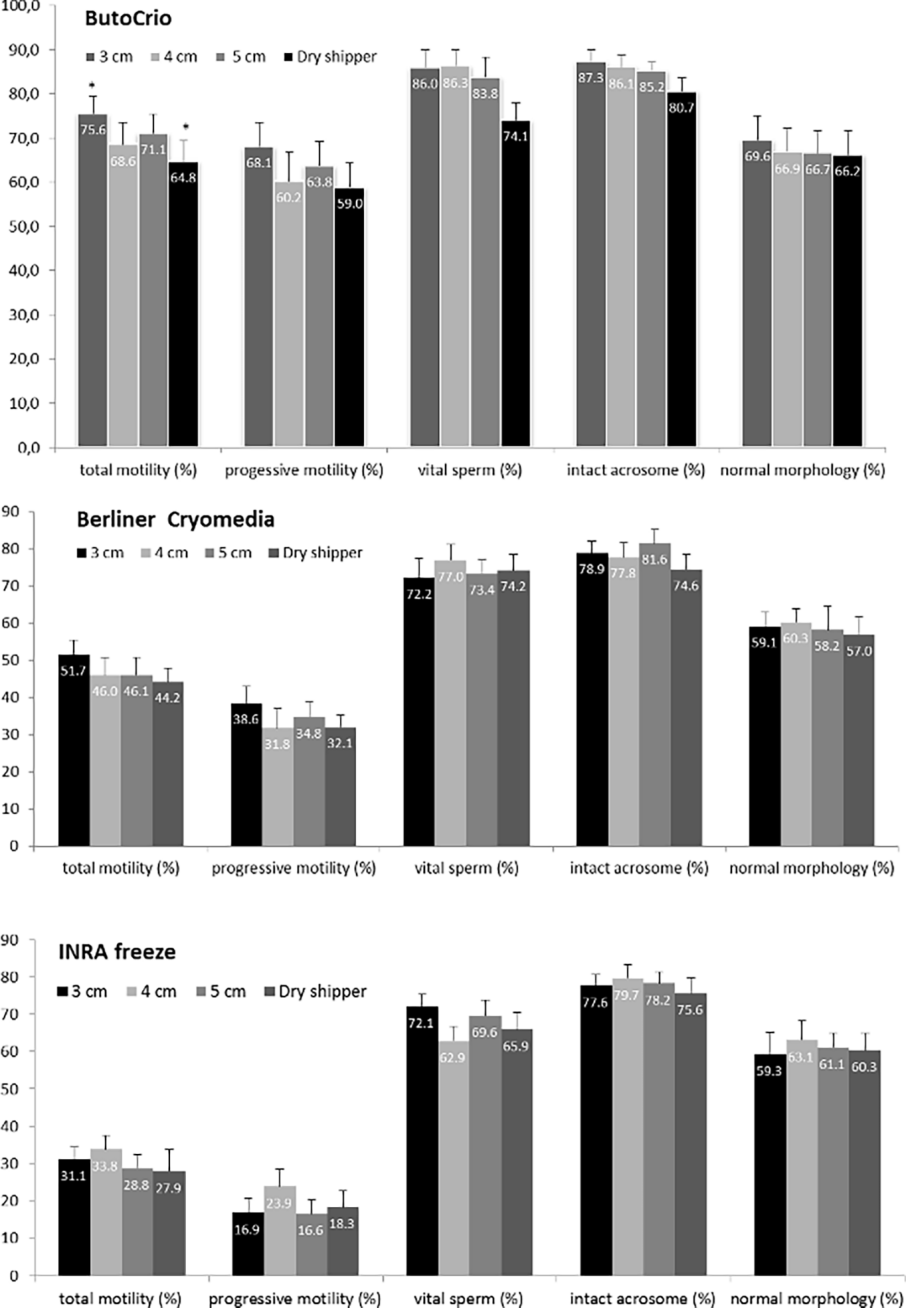
Post thaw sperm motility and morphology after thawing in rhinoceros (n = 9). Post thaw results for sperm frozen at a distance of 3, 4, 5 cm above the LN surface or in a dry shipper are compared between ButoCrio, Berliner Cryomedia and INRA Freeze.

**Fig 2:**
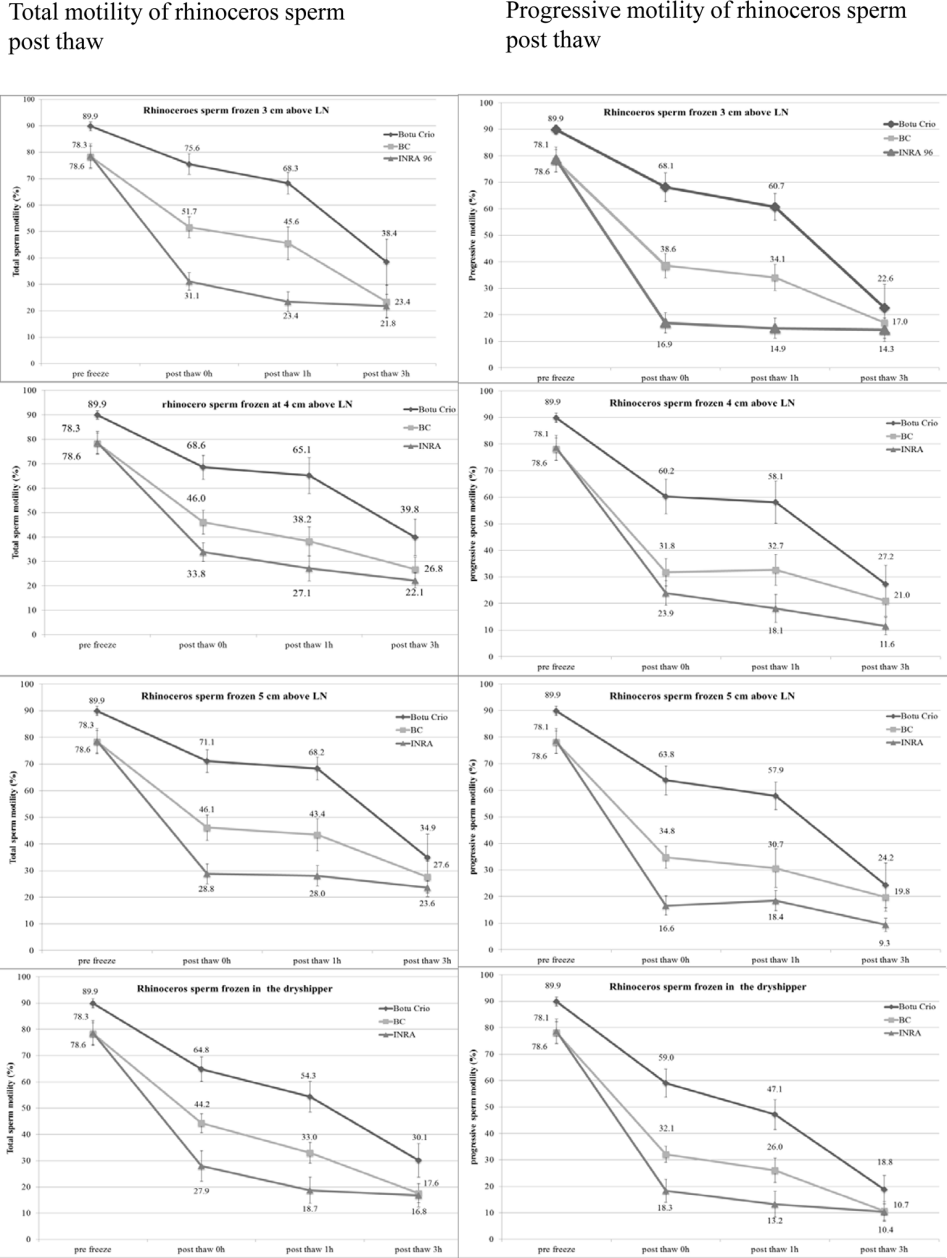
Development of motility of frozen thawed rhinoceros sperm cryopreserved at different freezing rates over a 3 h temperature challenge at 36.5°C.

Rhinoceros sperm was best cryopreserved when diluted with ButoCrio containing glycerol and methylformamide as cryoprotectants and frozen at a fast freezing rate 3cm above LN vapour (Fig 1). This variant produced superior mean post thaw results compared to sperm diluted in BC or INRA Freeze containing DMSO and glycerol, respectively. Post thaw total motility, progressive motility, sperm vitality and acrosome integrity of 75.6 ± 3.9%, 68.1 ± 5.4%, 86.0 ± 4.0% and 87.3 ± 2.6%, respectively were significantly higher compared to the other extenders at the same freezing rate (Fig 1, Table 2). Regardless if frozen at 3, 4, 5 cm over LN vapour or in the dryshipper, semen cryopreserved with BotuCrio had significantly higher total and progressive motility 0h and 1h after thawing when compared to BC and INRA Freeze at the respective freezing rate (p < 0.01–0.001) (Fig 2, Table 2,). Only after frozen-thawed spermatozoa had been temperature challenged at 36.5°C for 3 h differences in total and progressive motility between extenders became non-significant (Fig 2). Vitality of semen frozen with BotuCrio was also significantly higher when compared to semen cryopreserved with BC or INRA Freeze (p < 0.05–0.001), except for one variant. When frozen in the dryshipper post thaw sperm vitality of samples diluted with BotuCrio was not significantly different from those extended with BC (p > 0.05) (Table 2). Acrosome integrity of samples frozen with BotuCrio was significantly higher when cryopreserved at 3 cm and 4 cm above LN than samples frozen with BC or INRA Freeze (p < 0.05, Table 2). Samples frozen at 5 cm above LN or in the dryshipper showed no difference in acrosome integrity between BotuCrio and the other two extenders (p > 0.05). The post thaw sperm morphology showed no significant difference between the three extenders at any of the different freezing rates (p > 0.05). Most common abnormalities observed were detached sperm heads and tail defects.

**Table 2:** Significant differences between semen frozen with BotuCrio and BC or BotuCrio and INRA Freeze at four different freezing rates.

Sperm parameter	Significance of ButoCrio vs BC				Significance of ButoCrio vs INRA			
post thaw	3 cm	4 cm	5 cm	dryshipper	3 cm	4 cm	5 cm	dryshipper
Total sperm motility at 0 h	<0.001	<0.001	<0.01	<0.05	<0.001	<0.001	<0.001	<0.001
Total sperm motility at 1 h	<0.01	<0.001	<0.001	<0.001	<0.001	<0.001	<0.001	<0.001
Total sperm motility at 3 h	n.s.	n.s.	n.s.	n.s.	n.s.	n.s.	n.s.	n.s.
Progressive sperm motility at 0 h	<0.001	<0.001	<0.001	<0.01	<0.001	<0.001	<0.001	<0.001
Progressive sperm motility at 1 h	<0.001	<0.01	<0.01	<0.001	<0.001	<0.001	<0.001	<0.001
Progressive sperm motility at 3 h	n.s.	n.s.	n.s.	n.s.	n.s.	n.s.	n.s.	n.s.
Sperm vitality	<0.01	<0.05	<0.05	n.s.	<0.01	<0.001	<0.001	<0.05
Acrosome integrity	<0.05	<0.05	n.s.	n.s.	<0.05	<0.05	n.s.	n.s.
Normal morphology	n.s.	n.s.	n.s.	n.s.	n.s.	n.s.	n.s.	n.s.

n.s.: not significant

#### Effect of freezing rate on post thaw sperm quality

Rhinoceros semen was frozen at a distance of 3, 4, and 5 cm from the LN surface. In addition a forth variant was directly frozen in the dryshipper to mimic remote field conditions. None of these four freezing rates, either slow, fast or very fast in the dryshipper showed a significant difference in total or progressive sperm motility, vitality, acrosome integrity or normal morphology post thaw when compared within the same extender (p > 0.05) except for one variant (Fig 1). Samples frozen with BotuCrio at 3 cm above LN had significantly higher total motility (p < 0.01) versus samples directly frozen in the dryshipper. Yet, all other semen parameters of this particular variant, progressive sperm motility, vitality, acrosome integrity and normal morphology were not significantly different (Fig 1).

### Experiment II

#### Effect of removal of seminal plasma on post thaw sperm quality

Centrifugation of semen and removal of seminal plasma did not show a significant improvement of post thaw sperm quality compared to semen not centrifuged. None of the post thaw sperm parameters, total motility, progressive motility, acrosome integrity, sperm vitality or normal morphology, showed a significant difference between samples where seminal plasma had been removed and those it had remained (p > 0.05) (Table 3).

**Table 3:** Comparison of post thaw results (n = 5) when sperm was frozen with or without seminal plasma. Sperm was diluted with ButoCrio plus 1% glycerol + 4% methylformamide, frozen 3 cm above LN vapour.

Sperm parameter	Prior centrifugation	Post thaw	Post thaw	Significance
	& cooling	with seminal plasma	seminal plasma removed	(p < 0.05)
Total motility (%)	91.0 ± 1.8	68.6 ± 4.6	65.2 ± 5.4	n.s.
Progressive motility (%)	91.0 ± 1.9	58.0 ± 3.5	40.2 ± 12.6	n.s
Intact acrosomes (%)	90.4 ± 1.4	80.0 ± 4.4	77.6 ± 3.3	n.s
Normal morphology (%)	93.8 ± 1.7	85.6 ± 4.4	87.0 ± 4.3	n.s
Live sperm (%)	95.0 ± 1.8	91.4 ± 3.2	92.0 ± 1.7	n.s.

## Discussion

So far, there have been just three extensive studies on the cryopreservation of rhinoceros’ sperm from live donors using standard equine or similar extenders supplemented with either glycerol or DMSO. These studies have only reported comparatively moderate post thaw semen quality. This new, comprehensive study on sperm cryopreservation in three rhinoceros species is reporting substantially higher post thaw sperm motility compared to previous reports [13,15,17]. The mean post thaw sperm motility of 75.6 ± 3.9% reported above, is just 16% less than pre freeze motility and sets a new benchmark for male gamete preservation in rhinocerotidae.

The current body of knowledge on sperm cryopreservation in rhinoceros is based on just 9 publications since 1979 [10]. These publications comprised post thaw data from 73 semen samples from 50 animals of four rhinoceros species [11,12,13,14,15,16,17,29]. For comparison, a literature search on the Web of Science for publications on sperm cryopreservation in domestic bovine bulls listed 17 publications with 279 participating bulls in 2016 alone demonstrating rather minor advances in this area of research in rhinoceros. Yet, considering the overall limited data available on sperm cryopreservation in rhinoceros, progress over the past 40 years has been respectable but limited. Varying methods of semen collection (epidydimal preparation, electroejaculation, post coital collection) from live or deceased rhinoceros has been postulated as the reason for variable initial sperm quality and a large range of post thaw results. For example, in studies where epidydimal sperm was collected (n = 14 samples) [11,12,29] initial sperm quality was greatly influenced by previous chronic illness of the donor, elapsed time until the testis is extracted post mortem or the duration of chilled transport of the testis to the processing laboratory. These factors compromise initial sperm quality and subsequent cryopreservation results. As a consequence post thaw sperm quality in a recent study [29] did not surpass that of samples from live and healthy donors. As a consequence rescued and cryopreserved sperm may have only limited use in advanced assisted reproduction techniques such as IVF or ICSI.

In order to improve cryopreservation protocols for gamete rescue purposes in rhinoceros, we aimed to use only sperm-rich, high-quality sperm samples from live donors [23,24]. Sperm characterized by high progressive motility (90.3% ± 1.2%) and high sperm concentration (620 ± 102 x 10^6^ sperm / mL) was a prerequisite to test three cryomedia with different cryoprotectants each cryopreserved at four different freezing rates. Samples diluted with ButoCrio containing glycerol and methylformamide frozen 3 cm above LN vapour yielded the highest mean post thaw total (76%) and progressive (68%) sperm motility. All post thaw semen parameters of this variant except morphology, were significantly higher compared to the other cryomedia tested at the respective controlled freezing rates or when frozen at a very fast rate, mimicking remote field conditions. These results suggest that a short cooling time, a combination of glycerol and methylformamide as cryoprotectants and a fast freezing rate provide the best conditions for the cryopreservation of rhinoceros sperm.

The use of glycerol and DMSO as cryoprotectant agents of rhinoceros sperm, has been previously compared without showing any significant difference in post thaw sperm quality [12,17]. Their cell toxicity might be the reason for the fair but not exceptional freezability of rhinoceros sperm reported so far [15,17]. In general, glycerol toxicity is partly due to its high molecular weight and viscosity, resulting in slow cell membrane penetration and thus undesirable osmotic effects and cell dehydration. In contrast to glycerol, methylformamide has been reported to cause less osmotic damage to sperm because of lower molecular weight and viscosity [21]. Therefore, in the current study, we considered the combination of methylformamide and glycerol, reducing the cell toxic glycerol to 1% in the semen sample. We postulate this to be the main reason for the improved results in post thaw sperm quality compared to the other two diluents using glycerol or DMSO alone. It remains to be determined whether this increased sperm cryosurvial also results in higher pregnancy rates. Our results are in analogy to numerous reports in stallions, which showed improved post thaw sperm motility and higher pregnancy rate compared to other equine freezing media containing just glycerol [30,31,32]. Specifically in poor freezing stallions, the use of ButoCrio containing a combination of glycerol and methylformamide provided better post thaw motility as opposed to semen frozen with glycerol only [33]. Similarly to our study, shorter cooling times of 20–30 minutes to 5–8°C prior to freezing showed significantly improved post thaw sperm quality and better fertility in stallions with poor freezing spermatozoa [33,34].

Fundamental differences in extender composition might also have influenced the outcome of this study. For example, INRA Freeze produced the poorest post thaw results for rhinoceros sperm. Different from ButoCrio and BC, the egg yolk in INRA Freeze is replaced by egg yolk plasma and additional egg yolk phospholipids. It could be concluded that both the use of glycerol and the replacement of egg yolk by egg yolk plasma resulted in the poor cryosurvial of rhinoceros sperm. Similar conclusions are feasible for BC plus DMSO and ButoCrio plus glycerol and methylformamide. Further investigations are required to explore the true benefit of glycerol methylformamide over pure glycerol or DMSO formulations not only for the cryopreservation of rhinoceros sperm but for spermatozoa of other wildlife species.

The removal of seminal plasma is an integral part of sperm processing prior to cryopreservation and has resulted in improved post thaw quality in many domestic species such as the stallion, donkey and boar. Centrifugation of ejaculates and removal of seminal plasma has also been reported to be beneficial for the cryopreservation of sperm in wildlife species such as the Asian and the African elephant [22,28]. However, from the results of the current study, it appears that the same cannot be said for the rhinoceros species. The removal of seminal plasma did not further improve post thaw sperm quality and could therefore not be necessary for the cryopreservation of high quality samples. Our data also showed that centrifugation of rhinoceros sperm prior to freezing has no negative influence on post thaw sperm quality. As such, centrifugation and the use of a density gradients could be beneficial in wildlife breeding programs to remove dead or compromised sperm from poor semen donors or even remove cell debris after epidydimal sperm extraction to further improve ejaculate and post thaw sperm quality.

In light of the ongoing global poaching crisis and overall dismal outlook on the conservation status of all rhinoceros’ species, the value of captive assisted breeding programs is becoming increasingly important. The northern white rhinoceros is one such example, with only three living but infertile individuals left on the planet. This shows how earlier, improved systematic collection and cryopreservation of male and female gametes could have contributed to the current efforts to use IVF and embryo production to prevent this species from going extinct [19]. The new protocol for improved cryopreservation of rhinoceros’ sperm suggested in this study, helps by improving the quality of male rhinoceros’ gametes for long-term preservation, thus better preserving genetic diversity for the species and preventing future disasters.

## Supporting information

S1 Table Rhinoceros spermatozoa motility, morphology, acrosome integrity and viability before and after cryopreservation. (XLSX)
